# The Nuclear Protein HOXB13 Enhances Methylmercury Toxicity by Inducing Oncostatin M and Promoting Its Binding to TNFR3 in Cultured Cells

**DOI:** 10.3390/cells9010045

**Published:** 2019-12-23

**Authors:** Takashi Toyama, Sidi Xu, Ryo Nakano, Takashi Hasegawa, Naoki Endo, Tsutomu Takahashi, Jin-Yong Lee, Akira Naganuma, Gi-Wook Hwang

**Affiliations:** 1 Laboratory of Molecular and Biochemical Toxicology, Graduate School of Pharmaceutical Sciences, Tohoku University, Sendai 980-8578, Japan; takashi.toyama.c6@tohoku.ac.jp (T.T.); xusidi0606@hotmail.com (S.X.); ryo.nakano@case.edu (R.N.); t.hase.tohoku@gmail.com (T.H.); naoki.endou@mb.kyorin-pharm.co.jp (N.E.); tsutomu@toyaku.ac.jp (T.T.); leejy@dpc.agu.ac.jp (J.-Y.L.); naganuma@tohoku.ac.jp (A.N.); 2 Department of Environmental Health, School of Pharmacy, Tokyo University of Pharmacy and Life Sciences, 1432–1, Horinouchi, Hachioji, Tokyo 192–0392, Japan; 3 Laboratory of Pharmaceutical Health Sciences, School of Pharmacy, Aichi Gakuin University, 1-100 Kusumoto-cho, Chikusa-ku, Nagoya 464-8650, Japan

**Keywords:** methylmercury, HOXB13, oncostatin M, TNF receptor 3

## Abstract

Homeobox protein B13 (HOXB13), a transcription factor, is related to methylmercury toxicity; however, the downstream factors involved in enhancing methylmercury toxicity remain unknown. We performed microarray analysis to search for downstream factors whose expression is induced by methylmercury via HOXB13 in human embryonic kidney cells (HEK293), which are useful model cells for analyzing molecular mechanisms. Methylmercury induced the expression of oncostatin M (OSM), a cytokine of the interleukin-6 family, and this was markedly suppressed by HOXB13 knockdown. OSM knockdown also conferred resistance to methylmercury in HEK293 cells, and no added methylmercury resistance was observed when both HOXB13 and OSM were knocked down. Binding of HOXB13 to the OSM gene promoter was increased by methylmercury, indicating the involvement of HOXB13 in the enhancement of its toxicity. Because addition of recombinant OSM to the medium enhanced methylmercury toxicity in OSM-knockdown cells, extracellularly released OSM was believed to enhance methylmercury toxicity via membrane receptors. We discovered tumor necrosis factor receptor (TNF) receptor 3 (TNFR3) to be a potential candidate involved in the enhancement of methylmercury toxicity by OSM. This toxicity mechanism was also confirmed in mouse neuronal stem cells. We report, for the first time, that HOXB13 is involved in enhancement of methylmercury toxicity via OSM-expression induction and that the synthesized OSM causes cell death by binding to TNFR3 extracellularly.

## 1. Introduction

Inorganic mercury, present in the natural environment, is metabolized by microorganisms and converted to methylmercury. In the aquatic environment, methylmercury accumulates in large carnivorous fish, such as tuna, through the food chain [1]. Excessive consumption of methylmercury-contaminated fish causes severe central nervous system injury, such as Minamata disease [2]. Recent epidemiological studies have suggested that excessive intake of methylmercury during pregnancy has an adverse effect on fetal neural development [3,4]. However, the molecular mechanisms underlying methylmercury-induced central nervous system injury are still poorly understood. 

Previously, we identified the homeobox protein B13 (HOXB13), a transcription factor, as an intracellular factor involved in the enhancement of methylmercury toxicity [5] and established its relationship to cytotoxicity caused by an oxidative stress inducer and a thiol group modifier [6]. HOXB13 is highly expressed in the developmental stages of mice, especially in the chorda and posterior mesoderm, and has been suggested to be involved in the formation of normal nerve tubes by the induction of apoptosis [7]. Although HOXB13 expression is probably regulated by the long noncoding RNA, HOXB13-AS, in the adult brain [8], its function in the central nervous system remains largely unknown. In contrast, HOXB13 is highly expressed in the skin and prostate as well as in some cancer cells in adults and is known to be involved in promoting carcinogenesis [9,10,11,12]. According to a proposed mechanism, HOXB13 binds to the promoter of the inflammatory cytokine interleukin 6 (IL-6) gene to induce its expression in breast cancer cells and exhibit anticancer drug resistance [13]. Recent studies using mouse brain spheroid cultures have reported methylmercury-induced IL-6 in astrocytes to have a protective effect on the neurotoxicity caused by methylmercury [14]. However, our in vivo study using mice showed methylmercury induces the expression of tumor necrosis factor receptor (TNF)-α, IL-1β, and IL-19, but not IL-6, in the brain [15,16]. Therefore, the downstream factor that is induced by methylmercury via HOXB13 and is involved in methylmercury toxicity remains unknown.

To elucidate the mechanisms involved in the enhancement of methylmercury toxicity by HOXB13, we searched for genes that were induced to express by methylmercury via HOXB13 using microarray analysis, and we examined the relationship between methylmercury toxicity and a gene that showed altered expression after HOXB13 knockdown.

## 2. Materials and Methods

### 2.1. Cell Culture and Transfection

Human embryonic kidney cells (HEK293 cells) were maintained in Dulbecco’s modified Eagle’s medium (DMEM) (Nissui Pharmaceutical Co. Ltd., Tokyo, Japan), supplemented with 10% heat-inactivated fetal bovine serum (FBS), 2 mmol/L L-glutamine, and antibiotics (100 IU/mL penicillin and 100 mg/mL streptomycin), in a humidified incubator with 5% CO_2_/95% ambient air at 37 °C. Before the experiment, 5 × 10^3^ cells were seeded into a 96-well plate or 2.5 × 10^5^ cells were seeded into a 6-well plate. siRNA transfection was performed by HiPerFect transfection reagent (Qiagen, Hilden, Germany), and plasmid DNA transfection was done by Lipofectamine 2000 (Invitrogen, Carlsbad, CA, USA), according to the manufacturer’s instructions. Mouse neural stem cells (C17.2 cells) were maintained in DMEM, supplemented with 10% FBS, 5% horse serum (Thermo Fisher Scientific, Waltham, MA, USA), 2 mmol/L L-glutamine, and antibiotics (100 IU/mL penicillin and 100 mg/mL streptomycin). For introducing siRNA, 3 × 10^3^ cells were seeded into a 96-well plate or 8 × 10^4^ cells were seeded into a 6-well plate. siRNA transfection was performed by Lipofectamine RNAiMAX reagent (Thermo Fisher Scientific). Sequences of siRNAs, used in this study, are listed in Figure S1. Knockdown efficiency of HOXB13 siRNA was confirmed in previous report [5,6]. Specificity of all siRNAs was predicted by manufactures.

### 2.2. Microarray Analysis 

Changes in gene expression were evaluated by microarray-based two-color gene expression analysis (Agilent Technologies, Santa Clara, CA, USA), according to the manufacturer’s protocol. This array is specific for 26,083 human genes in Entrez database. Briefly, ISOGEN II (Nippon Gene, Tokyo, Japan) was used for total RNA isolation from cells. The obtained RNA was reverse transcribed by PrimeScript^TM^ RT Reagent Kit (Takara, Shiga, Japan) using a T7 promoter primer. cRNA was synthesized from the cDNA using T7 RNA polymerase to incorporate cyanine 3-CTP or cyanine 5-CTP. The labeled cRNAs were purified using RNeasy Mini protocol for RNA cleanup (Qiagen). Thereafter, cRNAs were fragmented using 25× fragmentation buffer at 60 °C for 30 min. Two samples were hybridized to microarray slips according to the manufacturer’s instructions. The hybridized slips were scanned at 532 nm and 635 nm using an Agilent Technologies Microarray Scanner (Agilent Technologies, Santa clara, CA, USA). The intensity and ratio of the two fluorescent signals were analyzed using GenePix image analysis (Molecular devices, San Jose, CA, USA). Data represent the mean of 3 repeated experiments of control siRNA vs. control siRNA + methylmercury or HOXB13 siRNA vs. HOXB13 siRNA + methylmercury.

### 2.3. Measurement of Cell Viability

Cells were cultured in a 96-well plate 24 h before starting the exposure. Cell viability was determined by the alamarBlue assay (Biosource, Camarillo, CA, USA), according to the manufacture’s protocol. Fluorescence was measured using a Gemini XPS microplate spectrofluorometer (Molecular Devices, Sunnyvale, CA, USA) with excitation wavelength of 545 nm and emission wavelength of 590 nm. 

### 2.4. Measurement of mRNA Levels by Quantitative PCR

Total RNA was purified by ISOGEN II (Nippon Gene) according to the manufacturer’s instructions. cDNA was synthesized from 500 ng of total RNA using the PrimeScript^TM^ RT reagent kit with oligo dT primer (Takara). Quantitative PCR (qPCR) was performed using SYBR Premix Ex Taq (Takara) with a Thermal Cycler Dice (Takara). Fold changes in mRNA levels were determined, and mRNA levels were normalized to those of glyceraldehyde-3-phosphate dehydrogenase (GAPDH). Data are represented as relative mRNA levels with control as 1. Primers used for qPCR are listed in Figure S1.

### 2.5. Measurement of OSM Protein Levels in the Medium

HEK293 cells were cultured in a 6-well plate for 24 h and treated with methylmercury at indicated concentration and time course. Thereafter, the medium was collected and subjected to human OSM ELISA (RayBiotech, Peachtree Corners, GA, USA) according to the manufacturer’s protocol. Recombinant human OSM protein was purchased from ProSpec (Rehovot, Israel).

### 2.6. Immunocytochemistry

HEK293 cells were cultured on glass slip and fixed with 4% paraformaldehyde (PFA) for 10 min at 25 °C. After rinsing with PBS, the cells were incubated with 0.1% TritonX- and 10% FBS-containing PBS for 30 min, and with anti-HOXB13 antibody (Cell Signaling Technology, Danvers, MA, USA), diluted in PBS, for 3 h. After two 5-min washes with PBS, the samples were incubated with fluorophore-conjugated antibody (Alexa Fluor plus 555, Thermo Fisher Scientific, MA, USA), diluted in PBS, for 1 h. Mounting was performed with VECTASHIELD mounting medium, hard set with 4’,6-diamidino-2-phenylindole (DAPI) (Vector Laboratories Inc. Burlingame, CA, USA). Confocal microscopy (FV1000, Olympus, Tokyo, Japan) was used for image acquisition. Specificity of HOXB13 immunostaining was confirmed by GFP-tagged HOXB13 expressing cells and HOXB13 knockdown cells (Figure S4).

### 2.7. Measurement of Promoter Activity in OSM Gene

Genomic DNA of HEK293 cells was purified by Genomic DNA purification kit (Toyobo, Osaka, Japan) according to the manufacturer’s instructions. The promoter of human OSM gene (−2100 to + 1 from transcription starting point) was amplified from the genomic DNA by PCR and cloned into pGL4.17 vector (reporter plasmid for promoter activity of the OSM gene). The resulting plasmid was transfected into HEK293 cells by Lipofectamine 2000 (Invitrogen) and incubated for 24 h. Firefly luciferase mRNA levels were determined as representative of the promoter activity of OSM gene, instead of luciferase enzymatic activity, since methylmercury directly inhibited the enzymatic reaction under the present condition. 

### 2.8. DNA-Protein Pull-Down Assay

An aliquot (180 ng) of genomic DNA from HEK293 cells was used to prepare a biotinylated-OSM gene promoter probe. Briefly, template genome was amplified by PCR using KOD-Plus Neo (Takara) and the following primers for OSM gene promoter (−2100 to + 1 bp from transcription start point): forward, 5′-GGATGAGATCGGTGGCTGGG-3′ and reverse, 5′-GCTGGGTGCCCGTGCTCCGG-3′ ± biotin. The resulting PCR product was purified using Oligo clean and concentrator kit (Zymo Research, Irvine, CA, USA) according to the manufacturer’s protocol and used as an OSM gene promoter probe. The purified probe (0.8 pmol) was incubated with the nuclear fraction (50 µg) of cells in Tris-buffered saline (TBS) for 3 h, with rotation, at 4 °C. Thereafter, 30 µL of streptavidin-agarose (Sigma, St. Louis, MO, USA) were added and further incubated for 1 h at 4 °C. Vehicle or probe without biotin (800 pmol) was added to the above incubation and further rotated at 4 °C for 3 h. Centrifugation (2300× *g* at 4 °C for 2 min) was performed, and the pellet was washed with PBS five times. The remaining pellet was eluted with SDS sample buffer and subjected to SDS-PAGE.

### 2.9. Immunoblotting

Cells were subjected to lysis by Cell-LyEX MP buffer (Fujifilm-Wako, Osaka, Japan), supplemented with protease inhibitor cocktail (Roche, Indianapolis, IN, USA), according to the manufacturer’s instructions. Protein concentrations in the cell lysates were determined by DC protein assay kit (Bio-Rad, Hercules, CA, USA). Aliquots of lysates were subjected to SDS-polyacrylamide gel electrophoresis (SDS-PAGE). In case of immunoblotting, a serum-free medium was used for cell culture, and 900 µL of medium were collected, to which 100 µL of trichloroacetic acid (TCA) were added and mixed vigorously. After 30-min incubation on ice, the medium was centrifuged (15,000× *g*, 4 °C for 10 min) and supernatant discarded. Ice-cold acetone (500 µL) was added to the precipitate and the pellet washed at once. The pellet was suspended in 50 µL of 50 mM Tris-HCl (pH 8.8) and 50 µL of 2× SDS-sample buffer and completely dissolved by incubation at 95 °C for 5 min. The samples were then subjected to SDS-PAGE. The resulting gel was transferred to an Immobilon-P membrane (Millipore, Burlington, MA, USA) and stained with anti-HOXB13 antibody (Cell Signaling Technologies), anti-OSM antibody (Abcam, Cambridge, UK), anti-V5 tag antibody (Fujifilm-Wako), and anti-GAPDH antibody (Santa Cruz Biotechnology, Dallas, TX, USA). 

### 2.10. Construction of V5 Tag Fused to N-Terminus of TNFR3 (TNFR3-V5) and OSM-Expressing Plasmid and Its Mutants

cDNA from HEK293 cells was used as a template. Coding region of TNFR3 mRNA was amplified by PrimeSTAR (Takara) using the following primers: 5′-GGGAAGGTACCATGACAGCCATCATCAAAGAG-3′ (sense) and 5′-AAATAGTGGGTACTGACTGGTACCTTCCC-3′ (antisense). The resulting PCR product was ligated into pEF5V/FRT/V5-DEST vector using Kpn1 (Takara) and Ligation high (Toyobo). OSM-expressing plasmid (RC204277) was obtained from Origene (Rockville, MD, USA). Both plasmids were transformed and amplified by DH5α (Nippon Gene, Tokyo, Japan), and purified using FastGene Plasmid mini kit (Nippon Genetics, Tokyo, Japan). Deletion of the extracellular domain of TNFR3-V5 (TNFR3Δaa31 to 227, i.e., TNFR3Δex) was performed by inverse PCR using the following primers: 5′-CTGGCCGTTCTGCTGCCACT-3′ (sense) and 5′-GGGCTGCGATGCTGCCAGGA-3′ (antisense). Deletion of the OSM signal peptide that is required for its extracellular release (OSMΔaa2 to 25, i.e., OSMΔsig) was performed according to the above method using the following primers: 5′-GCGGCTATAGGCAGCTGCTCGAAAG-3′ (sense) and 5′-CATGGCGATCGCGGCGGCAGATCTC-3′ (antisense). Sequences of the above plasmids were confirmed by Sanger Sequencing (FASMAC, Kanagawa, Japan).

### 2.11. Immunoprecipitation

Twenty microliters of anti-V5-tag antibody bead (Fujifilm-Wako) slurry were used for the immunoprecipitation (IP) experiment. The slurry was centrifuged (8000× *g*, 4 °C for 1 min), and the supernatant removed. After washing twice with 1 mL TBS, 100 µg of cell lysate were added to it. After rotating for 2 h at 4 °C, the beads were collected by centrifugation (8000× *g*, 4 °C for 1 min). The obtained beads were washed thrice with TBS, followed by the addition of 40 µL of 2× SDS-sample buffer, and incubation at 95 °C for 5 min for elution and denaturation. The supernatant was finally subjected to SDS-PAGE according to the methods described above.

### 2.12. Statistical Analysis

Statistical significance was analyzed by one-way ANOVA and Tukey’s post hoc test.

## 3. Results 

### 3.1. HOXB13 is Involved in the Induction of RFPL4A and OSM Expression by Methylmercury

**Figure 1:**
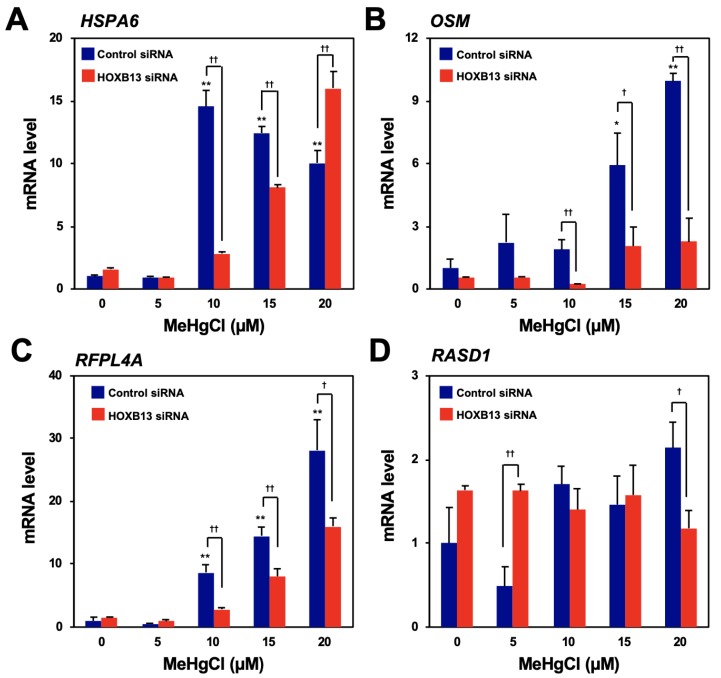
Confirmation of the induction of gene expression by methylmercury via HOXB13. Human embryonic kidney cells (HEK293) cells were transfected with control siRNA or HOXB13 siRNA for 48 h and exposed to indicated concentration of methylmercuric chloride (MeHgCl) for 6 h. Quantitative PCR (qPCR) for the top four genes listed in Table 1 ((**A**) HSP6A, (**B**) OSM, (**C**) RFPL4A, and (**D**) RASD1) was performed. Representative data indicate relative values, with control as 1, normalized to each glyceraldehyde-3-phosphate dehydrogenase (GAPDH) mRNA level. The values are presented as mean ± S.D. of three individual experiments. ^*^ *p* < 0.05 vs. control, ^**^ *p* < 0.01 vs. control, ^†^ *p* < 0.05 vs. control siRNA, ^††^ *p* < 0.01 vs. control siRNA.

To search for genes that are induced by methylmercury via HOXB13, microarray analysis was performed using the control and HOXB13-knockdown cells, untreated or treated with 20 µM methylmercuric chloride for 6 h, respectively. From the results, we identified 15 genes whose expression levels had increased four-fold or more after methylmercury treatment and whose elevated expression was significantly reduced by HOXB13 knockdown (Table 1). Among the above-mentioned genes, the top four genes (heat shock 70-kDa protein 6 [HSPA6], oncostatin M [OSM], ret finger-like protein 4A [RFPL4A], and dexamethasone-induced Ras-related protein 1 [RASD1]) with the highest rate of suppression by HOXB13 knockdown were selected, and their expression was confirmed by quantitative PCR. While the expression of HSPA6, RFPL4A, and OSM increased after treatment with methylmercury in a dose-dependent manner, that of RASD1 did not (Figure 1A–D). In addition, induction of RFPL4A and OSM expression by methylmercury was found to be significantly reduced through HOXB13 knockdown, although, conversely, HSPA6 expression increased after HOXB13 knockdown, in the presence of 20 µM methylmercuric chloride (Figure 1A–C). Therefore, at least HOXB13 was considered to be involved in the induction of RFPL4A and OSM expression by methylmercury.

HEK293 cells were transfected with control siRNA or HOXB13 siRNA for 48 h and exposed to 20 µM of methylmercuric chloride (MeHgCl) for 6 h. Methylmercury-induced gene expressions were shown as MeHgCl treated/control. The values are represented as mean ± S.D. (n = 3).

### 3.2. Induction of OSM Expression via HOXB13 Enhances Methylmercury Toxicity

**Figure 2:**
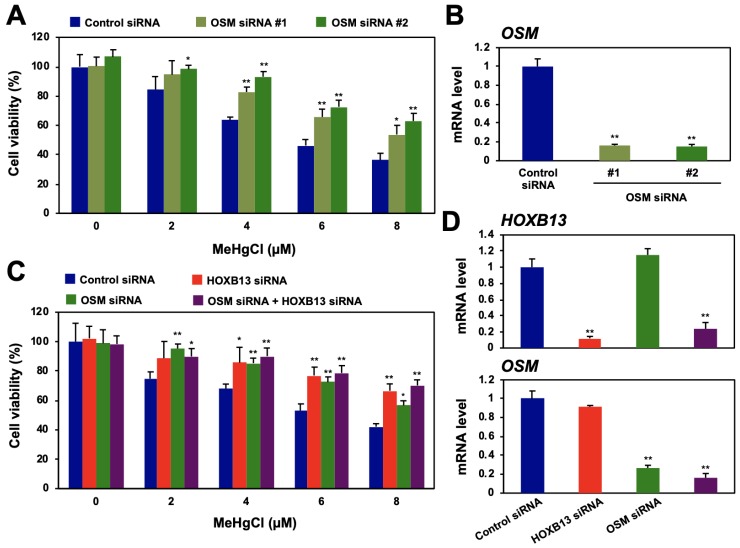
Effects of oncostatin M (OSM) and HOXB13 knockdown on cytotoxicity caused by methylmercury. (**A**) HEK293 cells were transfected with control siRNA or two different sequences of OSM siRNA (#1 or #2) for 48 h and exposed to indicated concentrations of methylmercuric chloride (MeHgCl) for 24 h. Cell viability was measured by alamarBlue assay. (**B**) qPCR for OSM mRNA was performed. Representative data indicate relative values, with control as 1, normalized to each GAPDH mRNA level. (**C**) Cells were transfected with control siRNA, OSM siRNA (#2), and/or HOXB13 siRNA for 48 h and exposed to indicated concentrations of MeHgCl for 24 h. Cell viability was measured by alamarBlue assay. No significant difference was found between HOXB13 siRNA and HOXB13 siRNA + OSM siRNA. (**D**) Control cells (without methylmercury exposure) were harvested, and qPCR for OSM mRNA (upper panel) or HOXB13 mRNA (lower panel) was performed. Representative data indicate the relative values, with control as 1, normalized to each GAPDH mRNA level. All values are mean ± S.D. of three individual experiments. ^*^ *p* < 0.05 vs. control siRNA, ^**^ *p* < 0.01 vs. control siRNA.

We next examined the effects of RFPL4A or OSM knockdown on HEK293-cell-sensitivity to methylmercury. When two different siRNA sequences for the RFPL4A gene were introduced, cell proliferation was strongly suppressed, and RFPL4A knockdown alone did not confer methylmercury resistance to the cells (Figure S2A,B). In contrast, the cells into which two different siRNA sequences for the OSM gene were introduced showed methylmercury resistance relative to control cells (Figure 2A,B). However, when OSM siRNA was introduced into HOXB13-knockdown cells already possessing methylmercury resistance, no further acquisition of resistance was observed (Figure 2C,D). We also confirmed that 4 and 8 µM of methylmercury induced OSM expression for 8–24 h, and this induction was suppressed by HOXB13 siRNA (Figure S3). Together, these results suggest that HOXB13 and OSM may be involved in methylmercury toxicity via the same pathway and that OSM is involved in the enhancement of methylmercury toxicity by HOXB13.

### 3.3. Methylmercury Promotes Binding of HOXB13 to the OSM Gene Promoter

**Figure 3:**
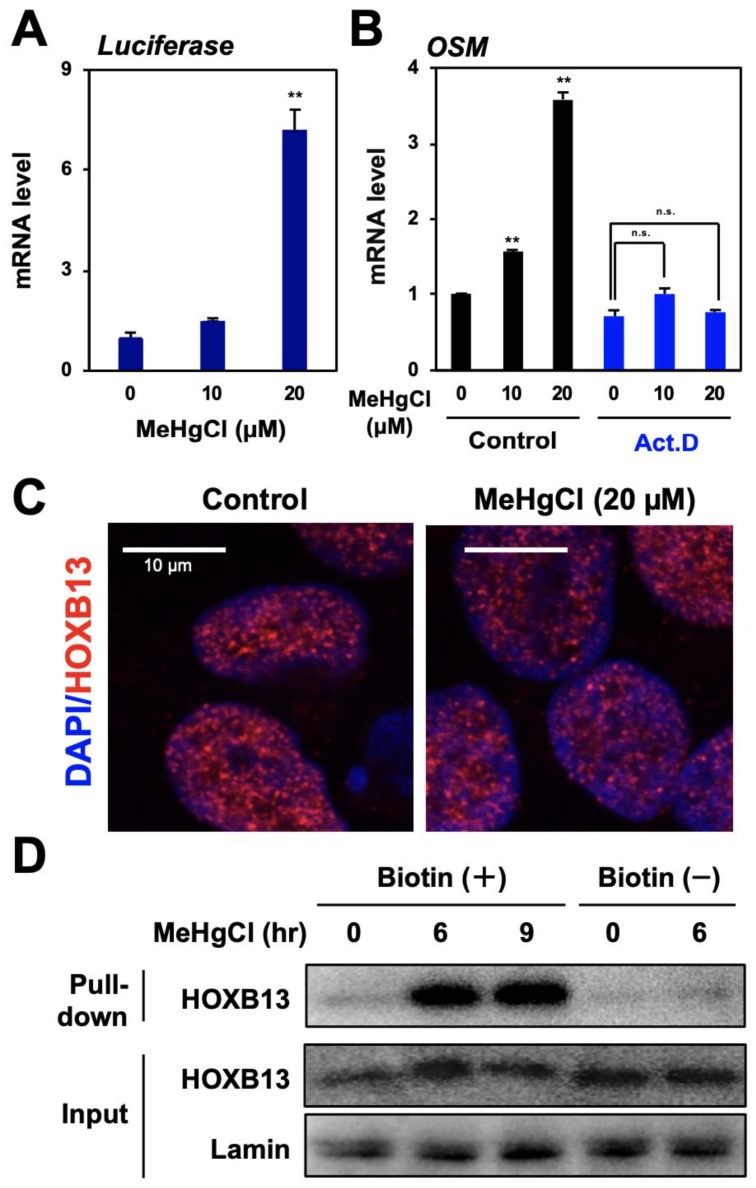
Role of HOXB13 in OSM expression induced by methylmercury. (**A**) HEK293 cells were transfected with a reporter plasmid for OSM gene promoter activity for 24 h and exposed to methylmercuric chloride (MeHgCl) for 6 h. A qPCR for firefly luciferase mRNA was performed. Representative data indicate the relative values, with control as 1, normalized to each GAPDH mRNA level. (**B**) Cells were pre-incubated with actinomycin D (Act. D) for 30 min and exposed to the indicated concentration of MeHgCl for 6 h. A qPCR for OSM mRNA was performed. Representative data indicate the relative values, with control as 1, normalized to each GAPDH mRNA level. (**C**) The cells were exposed to MeHgCl for 6 h, and immunostaining for HOXB13 was performed. Red indicates HOXB13, and blue indicates DAPI. Scale bar indicates 10 µm. (**D**) Cells were exposed to MeHgCl (20 µM) for indicated time course, and nuclear fractions were purified and subjected to a DNA–protein pull-down assay. All values are represented as mean ± S.D. of three individual experiments. ^**^ *p* < 0.01 vs. control siRNA, n.s. indicates not significant.

A reporter plasmid for the OSM gene promoter activity was introduced into HEK293 cells, and the promoter activity was measured using luciferase mRNA levels as an indicator. The results show that methylmercury treatment significantly increased luciferase mRNA levels (Figure 3A). Moreover, because induction of OSM expression by methylmercury was almost abolished by pretreatment with a transcription inhibitor (Figure 3B), methylmercury was considered to increase OSM mRNA levels through the promotion of its transcription. As described above, HOXB13 is known as a transcription factor with a homeobox domain, necessary for binding to DNA; however, there has been no report of the involvement of HOXB13 in the induction of OSM expression as a transcription factor. Under normal conditions, HOXB13 is mostly localized to the nucleus, and its distribution was unchanged even after treatment with methylmercury (Figure 3C). In contrast, when a DNA–protein binding assay was performed, using a probe in which biotin was added to the promoter region of OSM gene, binding of HOXB13 to the promoter of OSM gene was hardly observed under normal conditions, whereas it was significantly increased after treatment with methylmercury (Figure 3D). Incidentally, this binding almost disappeared when an excess of OSM promoter probe without a biotin tag was added. Taken together, these results suggest that methylmercury promotes the transcription of the OSM gene by increasing the binding of HOXB13 to its promoter.

### 3.4. TNF Receptor May Be Involved in the Enhancement of Methylmercury Toxicity by Extracellular OSM

**Figure 4:**
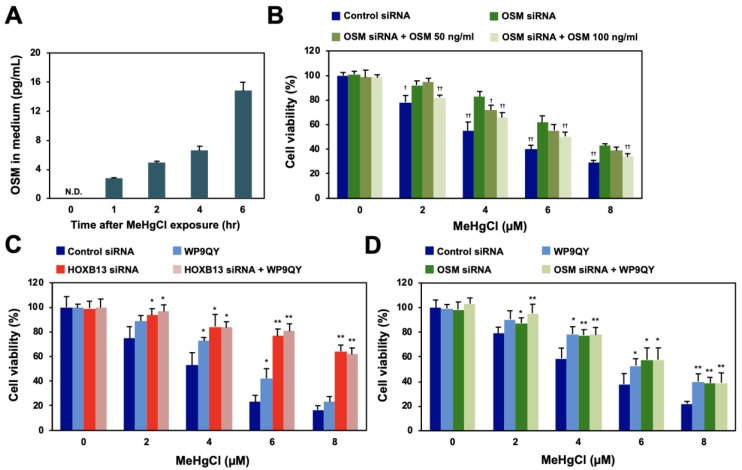
Effects of methylmercury on extracellular release of OSM and of the TNF receptor inhibitor on HOXB13/OSM-mediated methylmercury toxicity. (**A**) HEK293 cells were treated with 20 µM of methylmercuric chloride (MeHgCl) for the indicated time course, and OSM protein in the medium was measured by ELISA. (**B**) The cells were transfected with OSM siRNA (#2), and recombinant OSM was added to the medium. After incubation with MeHgCl for 24 h, cell viability was measured by alamarBlue assay. (**C**,**D**) The cells were transfected with HOXB13 siRNA (C) or OSM siRNA (D) for 48 h. WP9QY (10 µM), a TNF, was added to the medium 90 min before exposure to MeHgCl for 24 h. No significant difference was found between HOXB13 siRNA and HOXB13 siRNA + WP9QY or between OSM siRNA and OSM siRNA + WP9QY. All values are presented as mean ± S.D. of three individual experiments. ^*^ *p* < 0.05 vs. control siRNA, ^**^ *p* < 0.01 vs. control siRNA. ^†^ *p* < 0.05 vs. MeHgCl-treated control siRNA, ^††^ *p* < 0.01 vs. MeHgCl-treated control siRNA. N.D. indicates not detected.

**Figure 5:**
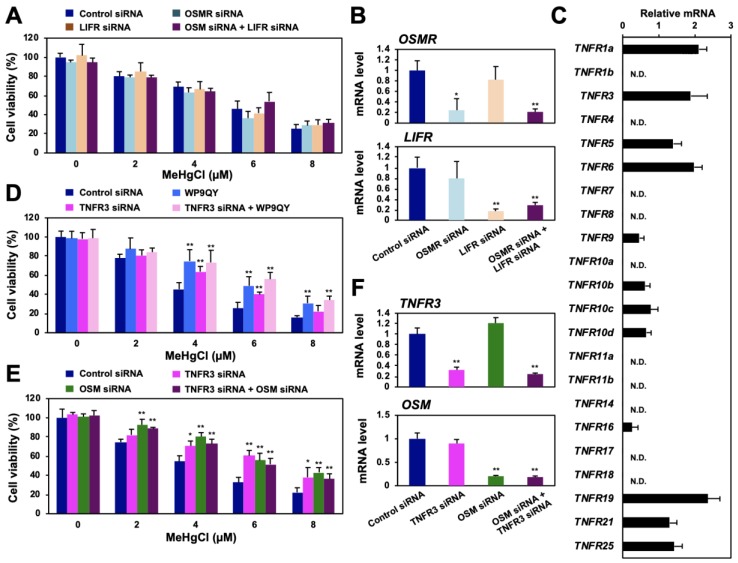
Identification of the TNF receptor associated with methylmercury toxicity via HOXB13 and OSM. (**A**) HEK293 cells were transfected with control siRNA, OSMR siRNA, and/or LIF receptor (LIFR) siRNA for 48 h and exposed to the indicated concentrations of methylmercuric chloride (MeHgCl) for 24 h. Cell viability was measured by alamarBlue assay. (**B**) Quantitative PCR for OSMR mRNA (upper panel) or LIFR mRNA (lower panel) was performed. Represented data indicate relative values, with the control as 1, normalized to each GAPDH mRNA level. (**C**) mRNA levels of TNF receptors in HEK293 cells were determined by qPCR. (**D**) Cells were transfected with TNFR3 siRNA (#2) for 48 h. WP9QY (10 µM) was added to the medium 90 min before exposure to methylmercuric chloride (MeHgCl) for 24 h. No significant difference was found between WP9QY and TNFR3 siRNA + WP9QY. (**E**) Cells were transfected with TNFR3 siRNA (#2) and/or OSM siRNA (#2) for 48 h. Cells were exposed to MeHgCl for 24 h, and cell viability was measured by alamarBlue assay. No significant difference was found between OSM siRNA and TNFR3 siRNA + OSM siRNA. (**F**) Control cells (without methylmercury exposure) were harvested, and qPCR for OSM (upper panel) or HOXB13 (lower panel) was performed. Representative data indicate the relative values, with control as 1, normalized to each GAPDH mRNA level. All values are presented as mean ± S.D. of three individual experiments. ^*^ *p* < 0.05 vs. control siRNA, ^**^ *p* < 0.01 vs. control siRNA. N.D. indicates not detected.

OSM is a cytokine belonging to the IL-6 family that is secreted extracellularly to exert various physiological activities, such as cell death induction, cell growth suppression, and bone regeneration [17,18,19]. When OSM was released into the culture medium of methylmercury-treated HEK293 cells, its levels were found to have increased in a time-dependent manner as a result of methylmercury treatment (Figure 4A). Although recombinant OSM was added to the culture medium of HEK293 cells, it did not affect cell death caused by methylmercury (data not shown). Therefore, considering the possibility that the endogenous OSM released extracellularly after methylmercury treatment was quantitatively sufficient to enhance methylmercury toxicity, recombinant OSM was added to OSM-knockdown cells. The sensitivity of the cells to methylmercury, after the addition of recombinant OSM, increased in a dose-dependent manner (Figure 4B), which suggests that methylmercury-triggered extracellularly released OSM is involved in cytotoxicity caused by methylmercury. The extracellularly released OSM is known to bind as a ligand to the OSM receptor (OSMR) or LIF receptor (LIFR) on the cell membrane [20,21]; however, knockdown of OSMR or/and LIFR did not affect methylmercury sensitivity (Figure 5A,B). We have previously shown that addition of WP9QY, a peptide that mimics the ligand-binding site of the TNF receptor (TNFR), to the medium suppresses methylmercury toxicity [15]. Therefore, the effects of WP9QY on the enhancement of methylmercury toxicity via HOXB13 were examined. The methylmercury-resistant HOXB13-knockdown cells showed almost no protective effect against methylmercury toxicity by WP9QY (Figure 4C), and, interestingly, a similar result was also obtained for the OSM-knockdown cells (Figure 4D). Together, these results suggest that, after being released extracellularly, OSM, which is induced by methylmercury via HOXB13, may cause cytotoxicity via binding to a certain TNF receptor that is inhibited by WP9QY.

### 3.5. OSM Enhances Methylmercury Toxicity via TNFR3

Although WP9QY was designed to mimic the ligand-binding site of TNFR1a, knockdown of this receptor did not affect the sensitivity of HEK293 cells to methylmercury (data not shown). WP9QY is also known to inhibit the binding of RANKL to TNFR11 (RANK) [22,23] and may have unexpectedly inhibited the binding of OSM to another TNFR. In humans, 29 types of TNFRs are known to exist [24], and we succeeded in measuring the mRNA of 22 human TNFRs by qPCR. Among them, expression of at least 12 types of TNFRs was observed in HEK293 cells (Figure 5C). Therefore, when siRNAs against these TNFRs were introduced into HEK293 cells to suppress TNFR expression, knockdown of TNFR3 or TNFR10c conferred methylmercury resistance to the cells (Figure S5A,B). When WP9QY was added to the knockdown cells, an additional increase in resistance to methylmercury was observed compared with that in the WP9QY-treated TNFR10c-knockdown cells (Figure S5C); in TNFR3-knockdown cells, the protective effect of WP9QY against methylmercury toxicity was not observed (Figure 5D). In addition, double knockdown of OSM and TNFR3 did not show any additional increase in resistance to methylmercury (Figure 5E,F). These results suggest that OSM may enhance methylmercury toxicity via TNFR3.

### 3.6. Methylmercury Promotes Binding of OSM to the Extracellular Domain of TNFR3

**Figure 6:**
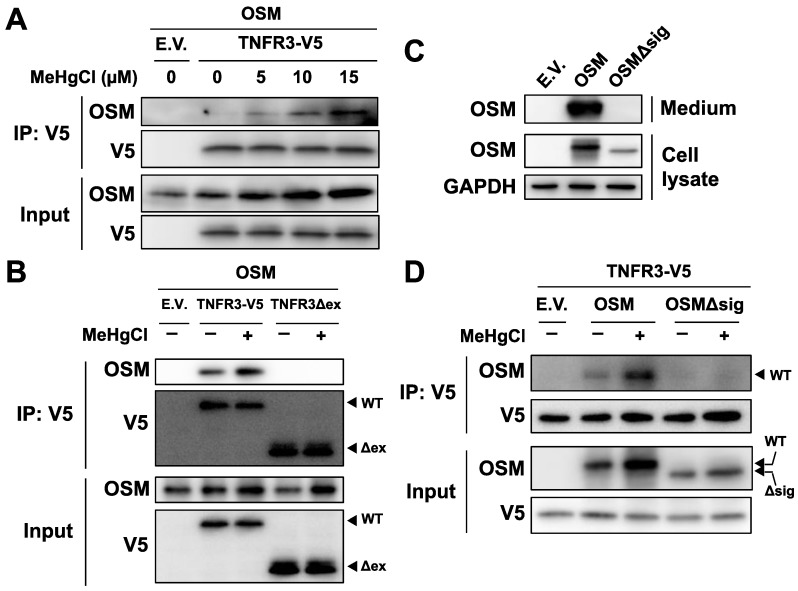
Effects of methylmercury on the binding between OSM and TNFR3. (**A**) HEK293 cells were transfected with TNFR3-V5- and/or OSM-expressing plasmids for 24 h and were exposed to methylmercuric chloride (MeHgCl) for 6 h. Cell lysates were subjected to immunoprecipitation (IP) by an antibody against V5-tag, and immunoblotting for OSM and V5 was performed. E.V. indicates an empty vector. (**B**) Cells were transfected with TNFR3- or TNFR3Δex- (deletion mutant of extracellular domain) and OSM-expressing plasmids for 24 h. Cells were exposed to MeHgCl (15 µM) for 6 h. IP by V5-tag was performed. (**C**) Cells were transfected with OSM- or OSMΔsig- (deletion mutant of the signal peptide required for extracellular release) expressing plasmids for 24 h. The incubation medium and cell lysates were subjected to immunoblotting for OSM. GAPDH was used as an internal control for cell lysates. (**D**) Cells were transfected with TNFR3- and OSM- or OSMΔsig-expressing plasmids for 24 h. IP by V5-tag was performed.

To confirm binding between OSM and TNFR3, plasmids expressing OSM and TNFR3-V5 were simultaneously introduced into HEK293 cells, and the obtained cell extracts were subjected to immunoprecipitation using beads that recognize the V5 tag. Under normal conditions, only a small degree of binding was observed between the two proteins; however, binding levels increased in a dose-dependent manner with methylmercury treatment (Figure 6A), indicating that TNFR3 may be a novel receptor to which OSM binds, and methylmercury increases its binding. Therefore, there is a possibility that OSM also binds to TNFR3 in cells. When a plasmid expressing mutant TNFR3 (TNFR3Δex), with the extracellular domain of TNFR3 deleted, was examined, similarly to as described above, no binding between OSM and TNFR3Δex was observed (Figure 6B). Moreover, a signal peptide sequence, necessary for extracellular release, was present at the N-terminus (amino acids 2–25) of OSM; a mutant OSM (OSMΔsig) lacking this sequence was not released into the medium (Figure 6C), and methylmercury could not increase its binding to TNFR3 (Figure 6D). From the above observations, methylmercury was revealed to promote the binding of extracellularly released OSM to the extracellular domain of TNFR3.

### 3.7. HOXB13 Enhances Methylmercury Toxicity via OSM/TNFR3 Pathway in Mouse Neural Stem Cells

**Figure 7:**
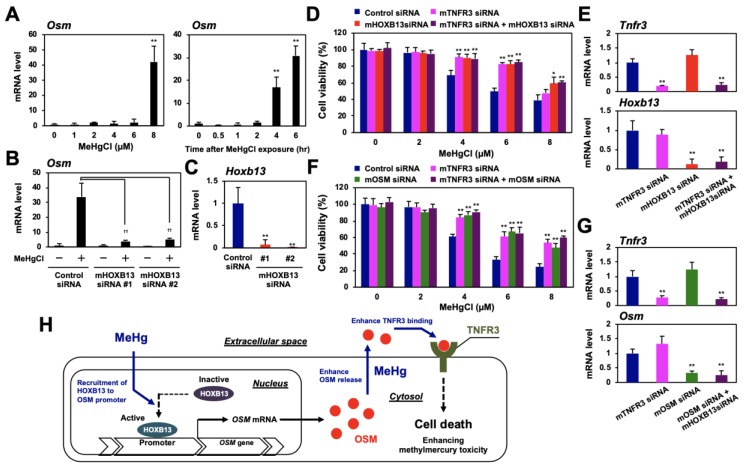
Involvement of HOXB13, OSM, and TNFR3 in methylmercury toxicity in mouse neural stem cells C17.2. (**A**) C17.2 cells were exposed to the indicated concentrations of methylmercuric chloride (MeHgCl) for 6 h (left panel) or to 8 µM MeHgCl for the indicated time course (right panel). OSM mRNA levels were determined by qPCR. Representative data indicate relative values, with control as 1, normalized to each GAPDH mRNA level. (**B**,**C**) Cells were transfected with two different sequences of mouse (m) HOXB13 siRNAs (#1 or #2) for 48 h. Cells were exposed to MeHgCl (8 µM) for 6 h, and mRNA levels of OSM and HOXB13 were determined. Representative data indicate the relative values, with control as 1, normalized to each GAPDH mRNA level. (**D**) Cells were transfected with control siRNA, mTNFR3 siRNA, and/or mHOXB13 siRNA (#2) for 48 h and exposed to indicated concentrations of MeHgCl for 24 h. Cell viability was measured by alamarBlue assay. No significant difference was found between mHOXB13 siRNA and mTNFR3 siRNA + mHOXB13 siRNA. (**E**) Quantitative PCR for TNFR3 (upper panel) or HOXB13 (lower panel) was performed. Representative data indicate the relative values, with control as 1, normalized to each GAPDH mRNA level. (**F**) Cells were transfected with control siRNA, mTNFR3 siRNA, and/or mOSM siRNA for 48 h and exposed to the indicated concentrations of MeHgCl for 24 h. Cell viability was measured by alamarBlue assay. No significant difference was found between mTNFR3 siRNA and mTNFR3 siRNA + mOSM siRNA. (**G**) qPCR for TNFR3 (upper panel) or OSM (lower panel) was performed. Representative data indicate the relative values, with control as 1, normalized to each GAPDH mRNA level. All values are mean ± S.D. of three individual experiments. * *p* < 0.05 vs. control siRNA, ** *p* < 0.01 vs. control siRNA, ^††^ *p* < 0.01 vs. MeHgCl-treated control siRNA. (**H**) Suggested mechanism underlying methylmercury toxicity through the induction of OSM via HOXB13 and OSM binding to TNFR3.

Although methylmercury is an environmental pollutant that causes severe neuronal damage, the role of HOXB13, OSM, and TNFR3 in methylmercury toxicity to neuronal cells is still unknown. To date, we have used mouse neural stem cells, C17.2, as a model cell line to clarify the involvement of various inflammatory cytokines in methylmercury toxicity [15,25,26,27,28]. When C17.2 cells were treated with methylmercury, OSM expression was induced in a time-dependent and dose-dependent manner (Figure 7A), and this induction was suppressed by HOXB13 knockdown (Figure 7B,C). Moreover, knockdown of OSM, HOXB13, or TNFR3 also conferred methylmercury resistance to C17.2 cells. However, when both HOXB13 and TNFR3, or OSM and TNFR3, were knocked down simultaneously, we did not observe additional methylmercury resistance (Figure 7D–G). From the above, we consider HOXB13 to be involved in the enhancement of methylmercury toxicity in C17.2 cells via the OSM/TNFR3 pathway.

## 4. Discussion

This study revealed that methylmercury promotes the binding of HOXB13 to the OSM gene promoter and induces OSM expression. In addition, methylmercury enhances its own cytotoxicity by promoting the binding of extracellularly released OSM to its novel receptor, TNFR3 (Figure 7H). The present study had some limitations, as the experiments were performed only on cultured cells and used a forced expression system. The binding efficiency of endogenous OSM with endogenous TNFR3 is still controversial. In addition, a nonspecific action of OSM cannot be excluded because high concentrations of recombinant OSM are required for the enhancement of methylmercury toxicity (Figure 4). However, because this finding was also observed in mouse neural stem cells (C17.2), there is strong evidence that the OSM/TNFR3-mediated pathway of HOXB13 is an important molecular mechanism involved in central nervous system injury due to methylmercury. 

Previous studies have suggested that HOXB13 is involved in the progression of cancer, including prostate, breast cancers, and glioblastoma [9,13,29]. The expression levels and nuclear localization of HOXB13 have also been suggested to be predictive markers for bladder cancer [30,31]; however, the physiological function of HOXB13 in the brain remains completely unknown. Recently, when we examined HOXB13 expression in 10- to 11-week-old C57BL/6 mice using semiquantitative PCR, it was below the detection limit in the liver, kidney, and rectum, whereas HOXB13 expression was confirmed in the cerebrum and cerebellum (unpublished observation). Thus, HOXB13 is, at least, expressed in the adult mouse brain and could be associated with central nervous system injury caused by methylmercury. In contrast, OSM has been reported to be expressed in microglia and astrocytes [32,33], whereas TNFR3 is expressed in neural stem cells of the brain [34]. In our study, HEK293 and mouse neuronal stem cells (C17.2) were used as simple models to show that methylmercury induces OSM expression via HOXB13 and causes cell death through TNFR3 in an autocrine manner. However, further studies are required into the distribution of HOXB13, OSM, and TNFR3 in the mouse brain and the effect of knocking out each gene on neuronal toxicity caused by methylmercury in mice. Knockdown of HOXB13, OSM, and TNFR3 was significantly protective against methylmercury toxicity; however, the protection was partial. This indicates that methylmercury may also induce cytotoxicity via other pathways. Elucidation of these pathways will lead to a more complete understanding of the mechanisms underlying methylmercury toxicity.

As mentioned above, HOXB13 has been shown to have a homeobox domain necessary for binding to DNA and may be a transcription factor involved in the induction of IL-6 or Regulatory factor X (RFX) gene expression by binding to the respective promoter [13,35]. In addition, HOXB13 has also been reported to bind to the TTTTATDRS sequence present in the RFX gene promoter [35]. However, this sequence is not present in the OSM gene promoter; therefore, methylmercury-induced binding of HOXB13 to this promoter is thought to involve an unknown mechanism. HOXB13 is observed in the progression of various cancers, and its increase is deeply involved in its activation [36,37,38]. However, methylmercury did not affect HOXB13 protein levels (Figure 3D), and OSM expression did not increase, even when HOXB13 was overexpressed in HEK293 cells (data not shown). These results suggest that methylmercury activates HOXB13 without affecting its levels. Recently, when we separated the HEK293 cell lysates by blue native PAGE and performed immunoblotting using the HOXB13 antibody, HOXB13, with a molecular weight of 31 kDa, was observed at 200 kDa or more, along with a smear (data not shown). In Figure 3C, HOXB13 can be seen as dots in the nucleus and was predicted to form a macromolecular complex in the nucleus under normal conditions. Taken together, methylmercury may induce OSM expression by affecting the formation of the complex, which contains HOXB13. If the proteins of the complex that bind to HOXB13 in the nucleus can be identified, the molecular mechanisms involved in the induction of OSM expression via HOXB13 by methylmercury should be clarified.

As described above, methylmercury is thought to enhance its own cytotoxicity by promoting the binding of OSM to TNFR3 extracellularly. OSM is known to bind as a ligand to two receptors, OSMR and LIFR [20,21]. When OSM binds, both receptors form heterodimers with gp130, thereby promoting the recruitment of JAK and STAT to its intracellular domain and exerting various physiological activities by activating downstream signal transduction [39]. Gliomas express both the receptors, and binding of OSM to these receptors suppresses cell proliferation via activation of the JAK-STAT pathway [40]. OSMR is known to be expressed in osteosarcoma, and binding of OSM to OSMR enhances apoptosis through JAK-STAT and p53 [18]. OSM in the dorsal root ganglion is involved in the survival of nociceptive neurons by binding to OSMR [41], and this pathway has also been found to suppress hyperpolarization by glutamate in studies using primary neurons [42]. Thus, OSM has been suggested to have different effects on the various nerve cells of the central nervous system. A neutralizing antibody for OSM/OSMR had little effect on methylmercury-induced cell death, indicating that the binding site of OSM/OSMR or LIFR and OSM/TNFR3 may be different, and the neutralizing effect on OSM/TNFR3 was restricted (Figure S6). If the neurons of the brain that express TNFR3—a novel OSM receptor identified in this study—can be identified, the novel function of OSM in the central nervous system and the molecular mechanisms involved in methylmercury-induced neuronal toxicity may be clarified. Again, the OSM/TNFR3 pathway contributes to methylmercury-induced cell death, but its extent is partial. The OSM/TNFR3-independent pathways that are involved in methylmercury toxicity remain unknown and should be examined in future studies.

The present study showed that TNFR3 is a membrane receptor involved in methylmercury toxicity. Although lymphotoxin beta (LTB) and LIGHT (TNFSF14) are known as TNFR3 ligands, methylmercury did not induce their expression in HEK293 cells, C17.2 cells, or mouse brains [15]. This indicates that known TNFR3 ligands may not be involved in the enhancement of methylmercury toxicity. In contrast, there was no amino acid sequence in OSM similar to that in LTB or LIGHT and no amino acid sequence in TNFR3 similar to the extracellular domain of OSMR or LIFR. Furthermore, 2 LTB forms a heterotrimer with 1 LTA and LIGHT forms a homotrimer, both of which subsequently bind to TNFR3 [43]; however, there has been no report of OSM forming a complex with itself or other molecules. In this study, the results indicate that, when overexpressed, OSM and TNFR3 can bind to each other; however, whether methylmercury-induced endogenous OSM can bind TNFR3 is still unclear. A stoichiometric approach will be necessary to confirm OSM as a novel ligand of TNFR3. To date, the molecular mechanisms involved in promoting the binding of OSM and TNFR3 by methylmercury remain unclear; however, methylmercury, which binds to free cysteine residues in proteins, may promote binding between OSM and TNFR3 by directly reacting with them.

The binding of LTB or LIGHT as a ligand to TNFR3 has been reported to activate c-Jun N-terminal kinase (JNK), which is involved in the induction of cell death [43,44]. In addition, LIGHT is known to induce apoptosis by binding to TNFR3 in some cancer cells [45]. Treatment of HEK293 cells with methylmercury activated JNK, but this activation was observed even when TNFR3 was knocked down (data not shown). Moreover, treatment of C17.2 cells with methylmercury increased the levels of cleaved caspase 3, which is an indicator of apoptosis; however, this increase was also unaffected by TNFR3 knockdown (data not shown). Together, these findings suggest that enhancement of methylmercury toxicity via the OSM/TNFR3 pathway may be through a novel molecular mechanism, without involving the known mechanisms for the induction of cell death via TNFR3.

Recently, the adverse effects of methylmercury on fetuses, due to pregnant women eating a diet high in fish, are becoming a global concern. As described above, TNFR3 is expressed in neural stem cells of the brain, and HOXB13 is known to be highly expressed during the developmental stages of the mouse [34]. Therefore, our finding that methylmercury toxicity is enhanced by the activation of the OSM/TNFR3 pathway via HOXB13 may be important for methylmercury toxicity during fetal development and could benefit future research into its prevention.

## Figures and Tables

**Table 1:** Gene expression induced by methylmercury via homeobox protein B13 (HOXB13).

	MeHgCl Treated/Control		
Gene	Control siRNA	HOXB13 siRNA	Ratio (HOXB13 siRNA/ Control siRNA)
*HSPA6*	37.17 ± 3.59	6.57 ± 1.71	0.18
*OSM*	5.23 ± 0.43	1.26 ± 0.18	0.24
*RFPL4A*	24.76 ± 4.38	6.11 ± 2.36	0.25
*RASD1*	18.12 ± 2.95	5.84 ± 0.48	0.32
*EDN1*	5.41 ± 0.75	1.72 ± 0.36	0.32
*GOLGA6A*	6.79 ± 0.93	2.36 ± 0.27	0.35
*RGS2*	18.02 ± 2.67	7.89 ± 1.46	0.44
*NR4A2*	9.27 ± 0.82	4.62 ± 0.37	0.50
*TNF-α*	12.04 ± 2.54	6.55 ± 1.02	0.54
*NEXN-AS1*	10.12 ± 1.43	5.76 ± 0.19	0.57
*FRG2C*	9.04 ± 0.89	5.45 ± 0.96	0.60
*GADD45G*	6.63 ± 0.57	4.19 ± 1.17	0.63
*HSD17B7*	9.63 ± 1.21	6.54 ± 1.16	0.68
*MIAT*	9.50 ± 0.97	7.03 ± 0.59	0.74
*HSPH1*	5.95 ± 0.59	4.88 ± 0.34	0.82

## Supplementary Materials

The following are available online at https://www.mdpi.com/2073-4409/9/1/45/s1, Figure S1: Sequence of primers for quantitative PCR and siRNAs, Figure S2: Effects of knockdown of RFPL4A or OSM on cytotoxicity caused by methylmercury, Figure S3: Effects of knockdown of HOXB13 on methylmercury induced OSM expression, Figure S4: Validation of immuno-fluorescence (IF) of HOXB13 in HEK923 cells, Figure S5: Effects of knockdown of TNF receptors on cytotoxicity caused by methylmercury, Figure S6: Effects of neutralizing antibody against OSM on methylmercury induced cell death in C17.2 cells.

## Abbreviations

HOXB13: Homeobox protein B13; HEK293: Human embryonic kidney cells; OSM: Oncostatin M; TNFR3: Tumor necrosis factor receptor 3; MeHgCl: Methylmercuric chloride; IL-6: Interleukin 6; DMEM: Dulbecco’s modified Eagle’s medium; FBS: Fetal bovine serum; TBS: Tris-buffered saline; GAPDH: glyceraldehyde-3-phosphate dehydrogenase; DAPI: 4’,6-diamidino-2-phenylindole.
