# Supplementary material for: The Nuclear Protein HOXB13 Enhances Methylmercury Toxicity by Inducing Oncostatin M and Promoting Its Binding to TNFR3 in Cultured Cells
Source: Cells. 2019 Dec 23;9(1):45. doi: 10.3390/cells9010045 (PMC7017003; doi:10.3390/cells9010045)
Supplement: Supplementary file 1 [file cells-09-00045-s001.pdf]

Primers used in this study

Target			Seqence
Human GAPDH	Forward	(5'-3')	GCACCGTCAAGGCTGAGAAC
	Reverse	(5'-3')	TGGTGAAGACGCCAGTGGA
Human HOXB13	Forward	(5'-3')	CAGATGTGTTGCCAGGGAGAAC
	Reverse	(5'-3')	AGGCGTCAGGAGGGTGCT
Human OSM	Forward	(5'-3')	CTCCTGAGACTTGGTTTACCTG
	Reverse	(5'-3')	TGTGTCCTCAGCTCTGTCTA
Human RFPL4A	Forward	(5'-3')	TGAACCGACAGGGGAAGATTG
	Reverse	(5'-3')	CAGGGCTCGCAAACAGGAAT
Human HSPA6	Forward	(5'-3')	CAAGGTGCGCGTATGCTAC
	Reverse	(5'-3')	GCTCATTGATGATCCGCAACAC
Human RASD1	Forward	(5'-3')	AGCTGAGTATCCCGGCCAA
	Reverse	(5'-3')	CGATGGTAGGCGTGTAGGC
Human TNFR1a	Forward	(5'-3')	GAGAGGCCATAGCTGTCTGG
	Reverse	(5'-3')	CTTCCTTTGTGGCACTTGGT
Human TNFR1b	Forward	(5'-3')	GGCAAGTCCCTGACTCTCTG
	Reverse	(5'-3')	AGAAAAGGATGCTGGGTTT
Human TNFR3	Forward	(5'-3')	AAGGATTCGTGGTGCTCATC
	Reverse	(5'-3')	GAATCCTACCCAACCCCCTA
Human TNFR4	Forward	(5'-3')	CCTCAGAAGTGGGAGTGAGC
	Reverse	(5'-3')	CAGATTGCGTCCGAGCTATT
Human TNFR5	Forward	(5'-3')	GCAGGCACAAACAAGACTGA
	Reverse	(5'-3')	TCGTCGGGAAAATTGATCTC
Human TNFR6	Forward	(5'-3')	TCAGTACGGAGTTGGGGAAG
	Reverse	(5'-3')	ACCTGGAGGACAGGGCTTAT
Human TNFR7	Forward	(5'-3')	CCAGCATAGAAAGGCTGCTC
	Reverse	(5'-3')	GGGTTTGGAAGAGGATCACA
Human TNFR8	Forward	(5'-3')	CTTCTGGGTGATCCTGGTGT
	Reverse	(5'-3')	CATCAGTGGCTGGCTCATTA
Human TNFR9	Forward	(5'-3')	CACTCTGTTGCTGGTCCTCA
	Reverse	(5'-3')	ACAGGTCCTTTGTCCACCTG
Human TNFR10a	Forward	(5'-3')	ACAGCAATGGGAACATAGCC
	Reverse	(5'-3')	TGGTGCAGGGACTTCTCTCT
Human TNFR10b	Forward	(5'-3')	TGCAGCCGTAGTCTTGATTG
	Reverse	(5'-3')	TCCTGGACTTCCATTTCCTG
Human TNFR10c	Forward	(5'-3')	AAAGTTCCTGCACCATGACC
	Reverse	(5'-3')	TGGCACCAAATTCTTCAACA
Human TNFR10d	Forward	(5'-3')	TCCAATATGGGGCAGCTTAC
	Reverse	(5'-3')	CCTGCCTCAGCCTATCAAAG
Human TNFR11a	Forward	(5'-3')	GGTGCAGCCTCTAACTCCTG
	Reverse	(5'-3')	GTTTGAGACCAGGCTGGGTA
Human TNFR11b	Forward	(5'-3')	GGCAACACAGCTCACAAGAA
	Reverse	(5'-3')	CGGTAAGCTTTCCATCAAGC
Human TNFR14	Forward	(5'-3')	AGGAATGTCAGCACCAGACC
	Reverse	(5'-3')	TCACCTTCTGCCTCCTGTCT
Human TNFR16	Forward	(5'-3')	GTGGGACAGAGTCTGGGTGT
	Reverse	(5'-3')	AAGGAGGGGAGGTGATAGGA
Human TNFR17	Forward	(5'-3')	AGCAGGCGAAGTTCATTGTT
	Reverse	(5'-3')	GCAAGCATGCAACAAACTGT
Human TNFR18	Forward	(5'-3')	GAGTGGGACTGCATGTGTGT
	Reverse	(5'-3')	TGCAGTCTGTCCAAGGTTTG
Human TNFR19	Forward	(5'-3')	TCCGTGAGAACACACCACAT
	Reverse	(5'-3')	GCACCGTCCTCTTAAATCCA
Human TNFR21	Forward	(5'-3')	GTGAACAAGACCCTCCCAAA
	Reverse	(5'-3')	CAGCAGGAAAAGCACAATCA
Human TNFR25	Forward	(5'-3')	CACCCTTCTAGCACCTCCTG
	Reverse	(5'-3')	TCCATCACGTCGTAGAGCTG
Human LIFR	Forward	(5'-3')	TGGAACGACAGGGGTTCAGT
	Reverse	(5'-3')	GAGTTGTGTTGTGGGTCACTAA
Human OSMR	Forward	(5'-3')	ATGGCTCTATTTGCAGTCTTTCA
	Reverse	(5'-3')	CACCCAGATGACATTGGATGTT
Firefly luciferase	Forward	(5'-3')	CGCACATATCGAGGTGGACA
	Reverse	(5'-3')	GCAAGCTATTCTCGCTGCAC
Mouse GAPDH	Forward	(5'-3')	AACTTTGGCATTGTGGAAGG
	Reverse	(5'-3')	ACACATTGGGGGTAGGAACA
Mouse HOXB13	Forward	(5'-3')	TCTTGCCGAGTATCCAGGAG
	Reverse	(5'-3')	GGAGTTTCCGAAGGGTAGGTAG
Mouse OSM	Forward	(5'-3')	ATGCAGACACGGCTTCTAAGA
	Reverse	(5'-3')	TTGGAGCAGCCACGATTGG
Mouse TNFR3	Forward	(5'-3')	CCCCTTATCGCATAGAAAACCAG
	Reverse	(5'-3')	TGCATACCGCAAAGACAAACT

siRNAs used in this study

Target gene	siRNA Sense Seqence (5'-3') (complement is used for antisense)	Location
Negative control siRNA	AAUUCUCCGAACGUGUCACGU	–
Human HOXB13	UACGCUGAUGCCUGCUGUCAA	(CDS: 276-297)
Human OSM #1	CACUGAGAAGUGCACUUUA	(3'UTR: 1097-1115)
Human OSM #2	UAAAGUGCACUUCUCAGUG	(5'UTR: 87-105)
Human RFPL4A #1	CAACUGAGCCCAUGCUGAU	(3'UTR: 1771-1795)
Human RFPL4A #2	AUCAGCAUGGGCUCAGUUG	(5'UTR 67-85)
Human TNFR1a #1	ACCGGCAUUAUUGGAGUGAAA	(CDS: 676-686)
Human TNFR1a #2	ACGGUGGAAGUCCAAGCUCUA	(CDS: 992-1012)
Human TNFR3 #1	GAUGAAGUUGGGAAGGGUA	(3'UTR: 2038-2055)
Human TNFR3 #2	UACCCUUCCCAACUUCAUC	(5'UTR 121-139)
Human TNFR5 #1	ACAGAGUUCACUGAAACGGAA	(CDS: 231-251)
Human TNFR5 #2	UUGGUGCUGGUCUUUAUCAAA	(CDS: 643-663)
Human TNFR6 #1	AAGGACAUUACUAGUGACUCA	(3'UTR: 1267-1287)
Human TNFR6 #2	UUGAGGAAGACUGUUACUACA	(CDS: 455-475)
Human TNFR9 #1	CUGGUACAUUCUGUGAUAAUA	(CDS: 226-246)
Human TNFR9 #2	CAAGAACACCAUCCUACAUAA	(3'UTR: 979-999)
Human TNFR10b #1	CCGACUUCACUUGAUACUAUA	(3'UTR: 1885-1905)
Human TNFR10b #2	CUGGACAACCUUACAAGUAUA	(3'UTR: 2541-2561)
Human TNFR10c #1	ACCAACGCUUCCAACAAUGAA	(CDS: 427-447)
Human TNFR10c #2	AUCGUAGGGAUCAUAGUUCUA	(CDS: 934-954)
Human TNFR10d #1	CCGGAGUGACAUCAAGUGCAA	(CDS: 614-634)
Human TNFR10d #2	UAGAACGGGAUUAUCUUGUUA	(3'UTR: 1814-1834)
Human TNFR16 #1	AACGUUAAGUGAUGAACAUUA	(3'UTR: 3356-3376)
Human TNFR16 #2	AUGGCAAUUCUUUGACCUCAA	(3'UTR: 3033-3053)
Human TNFR19 #1	ACGAAACUUGUCGGCUUUCAA	(CDS: 437-457)
Human TNFR19 #2	UUGGAUUCAAAUAGCAGUCAA	(CDS: 1103-1123)
Human TNFR21 #1	CUGAGCAUUGUACCAACACAA	(CDS: 663-683)
Human TNFR21 #2	ACCGUGUAGAAUGCUUGAUUA	(3'UTR: 3082-3102)
Human TNFR25 #1	CACCGUCCAGUUGGUGGGUAA	(CDS: 769-789)
Human TNFR25 #2	CGCGGUAUUAAAUCUGUGAAA	(3'UTR: 1611-1631)
Human LIFR	UACGAAUCUUGAUUGCAAC	(3'UTR 2393-2411)
Human OSMR	GCAUGAAAGGCAUCGUUCU	(3'UTR: 5050-5068)
Mouse HOXB13 #1	GAUGUGUUGCCAAGGUGAA	(CDS: 672-691)
Mouse HOXB13 #2	GCUACCUACCCUUCGGAAA	(CDS: 446-465)
Mouse TNFR3	CUGUGACUGUCACCGGCAA	(CDS: 1237-1256)
Mouse OSM	CUGAUCCGGUGCUCUCUCU	(CDS: 1286-1305)

Figure S1. Sequence of primers for quantitative PCR and siRNAs.

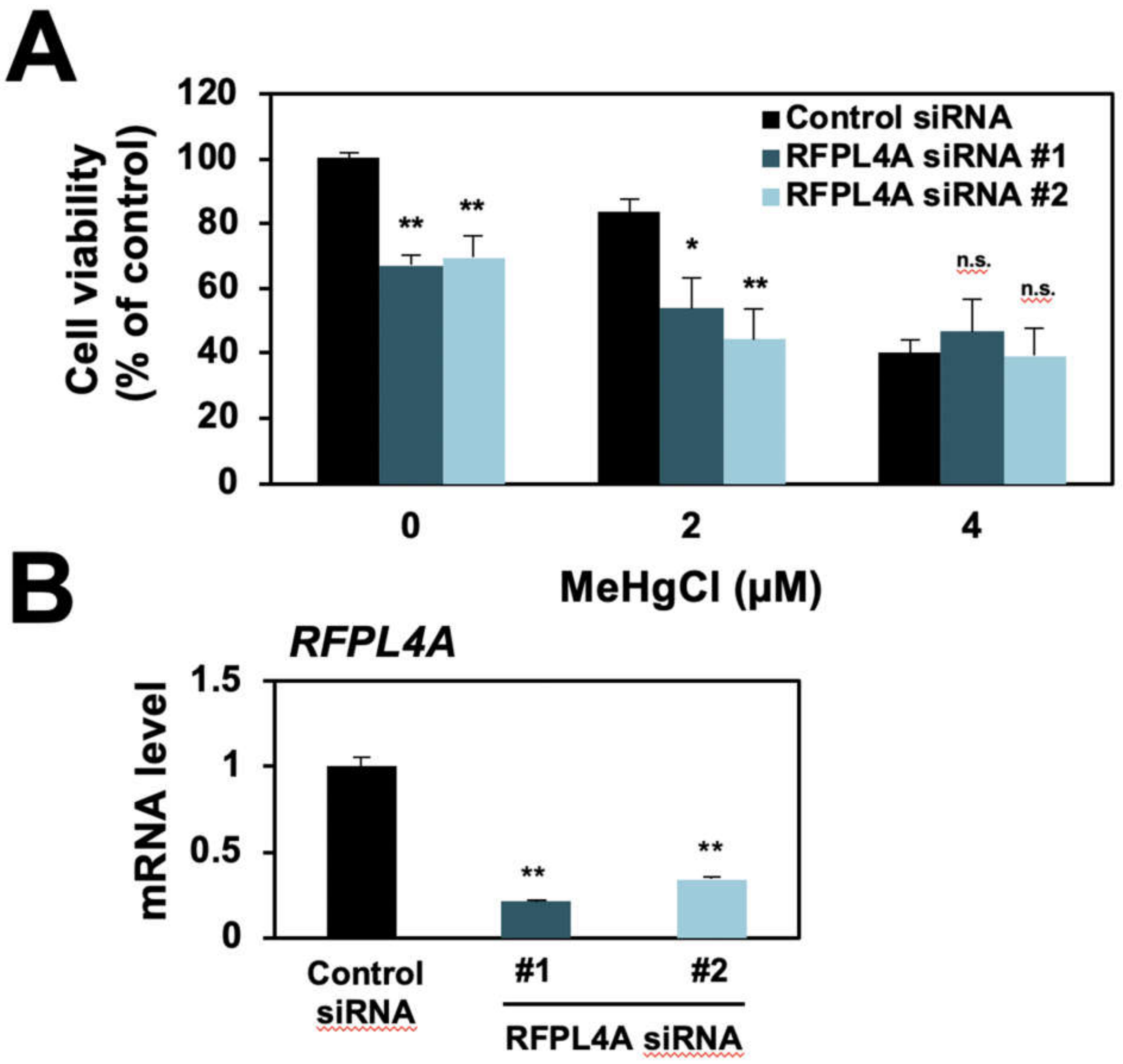

Figure S2. Effects of knockdown of RFPL4A or OSM on cytotoxicity caused by methylmercury. (A) HEK293 cells were transfected with 2 different sequence of RFPL4A siRNA (#1 or #2) for 48 h and cell viability was measured by alamarBlue assay. (B) Quantitative PCR for RFPL4A mRNA was performed. Represented data indicate relative value as control as 1, that normalized by each GAPDH mRNA level. All values are represented as mean ± S.D. (n=3). $^{*}P<0.05$ vs control siRNA, $^{**}P<0.01$ vs control siRNA. n.s. indicates not significant.

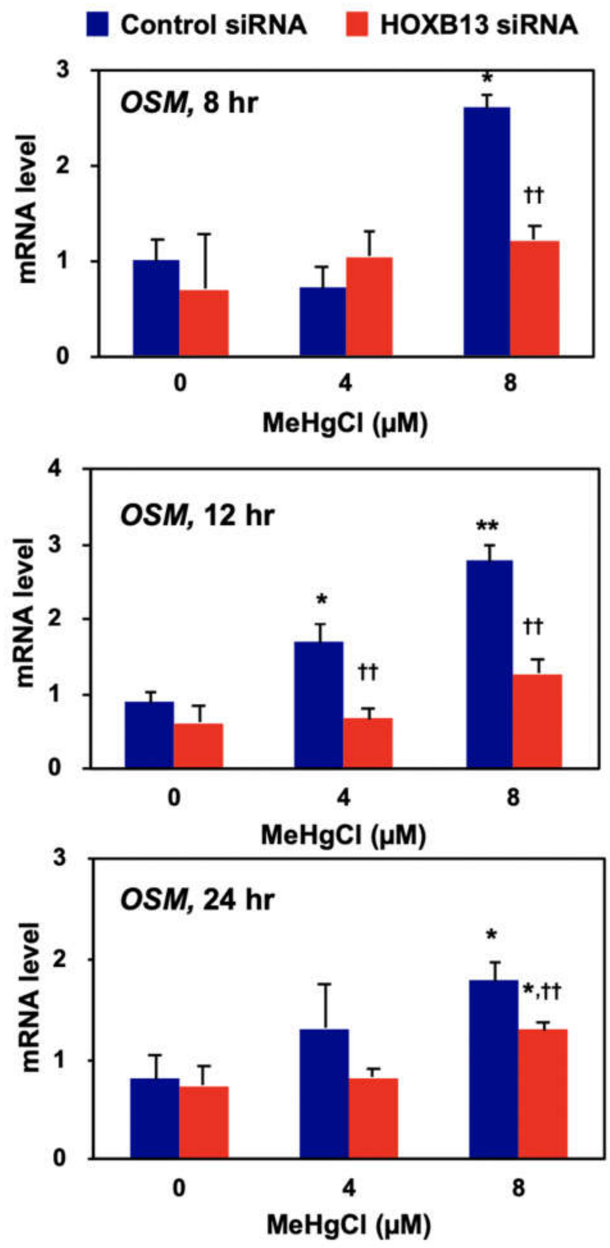

Figure S3. Effects of knockdown of HOXB13 on methylmercury induced OSM expression. (A) HEK293 cells were transfected with control siRNA or HOXB13 siRNA for 48 h and exposed to indicated concentrations of methylmercuric chloride (MeHgCl) for indicated time course. Quantitative PCR for OSM mRNA was performed. Represented data indicate relative value as control as 1, which normalized by each GAPDH mRNA level. All values are mean ± S.D. of 3 individual experiment. $^{*}P<0.05$ vs control (MeHgCl 0 μM), $^{**}P<0.01$ vs control (MeHgCl 0 μM). $^{\dagger}P<0.05$ vs Control siRNA, $^{\dagger\dagger}P<0.01$ vs Control siRNA.

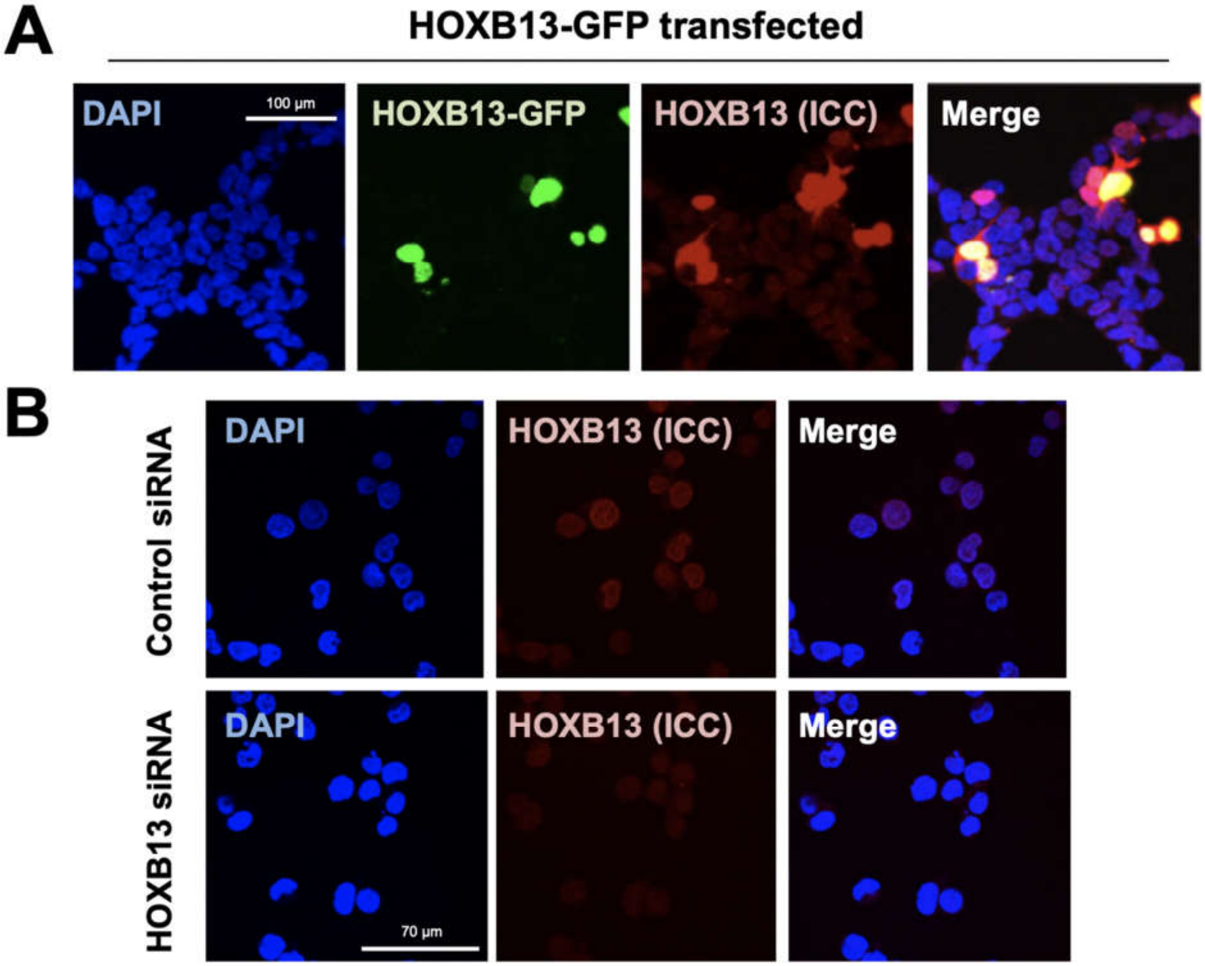

Figure S4. Validation of immuno-fluorescence (IF) of HOXB13 in HEK923 cells

HEK293 cells were seeded on glass slip and transfected with pEGFP-C2/human HOXB13 plasmid for 24 h (A) or HOXB13 siRNA for 48 h (B). Then IF was performed using HOXB13 antibody. Red indicates HOXB13, blue indicates DAPI and green indicates GFP. Scale bar indicates 70 μm.

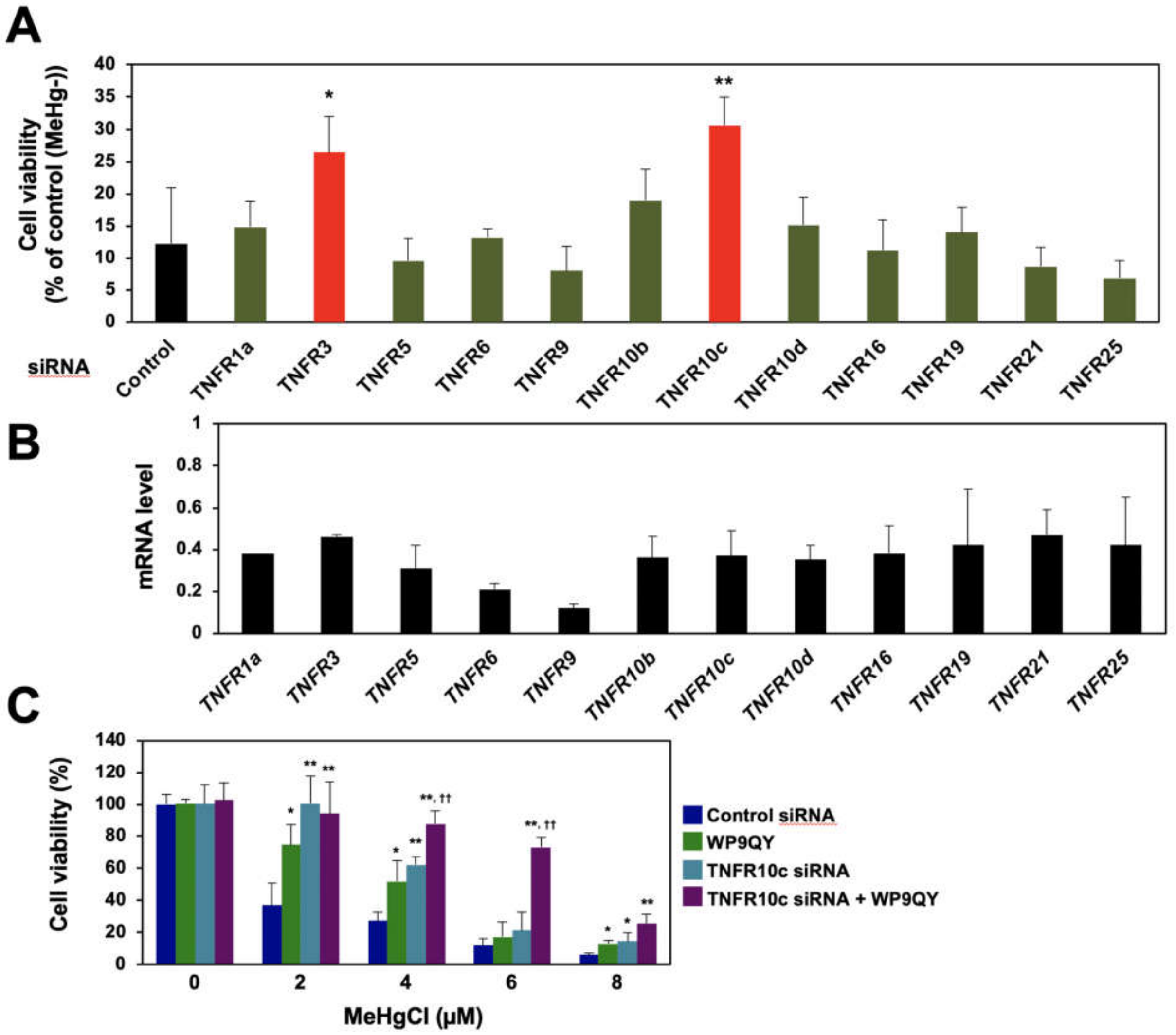

Figure S5. Effects of knockdown of TNF receptors on cytotoxicity caused by methylmercury. (A) HEK293 cells were transfected with indicated siRNA (equivalent mix of #1 and #2 siRNAs) for 48 h. The cells were exposed to 8 μM of methymercuryc chloride (MeHgCl) for 24 h and cell viability was measured by alamerBlue assay. The value of non-treated control cells was estimated as 100%. (B) mRNA level of siRNA transfected cells in (A) was measured by quantitative PCR. Represented data indicate relative value as each control as 1, which normalized by each GAPDH mRNA level. (C) The cells were transfected with TNFSF10c siRNA (equivalent mix of #1 and #2 siRNAs) for 48 h. WP9QY (10 μM) was added to the medium 90 min before the exposure of MeHgCl for 24 h. All values are represented as mean ± S.D. (n=3). $^{*}P<0.05$ vs control siRNA, $^{**}P<0.01$ vs control siRNA, $^{\dagger}P<0.05$ vs TNFRSF10c siRNA, $^{\dagger\dagger}P<0.01$ vs TNFRSF10c siRNA.

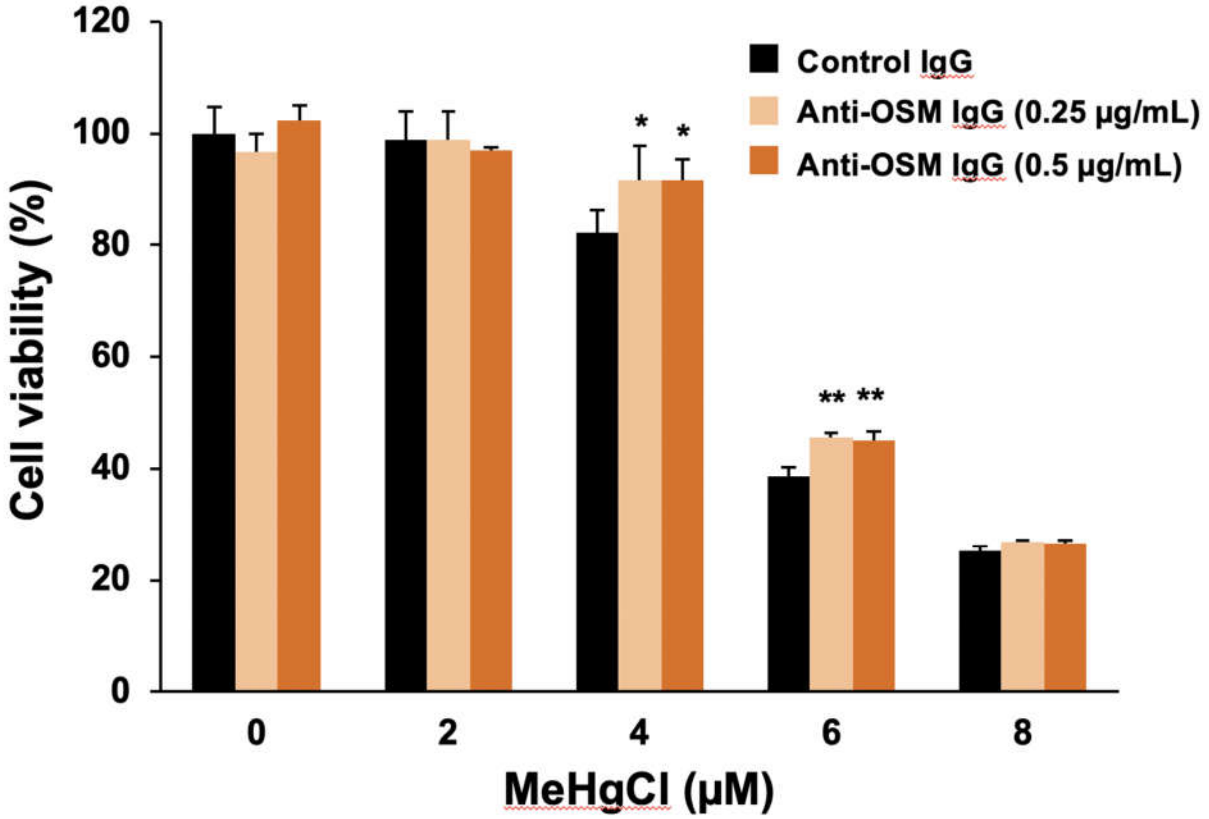

Figure S6. Effects of neutralizing antibody against OSM on methylmercury induced cell death in C17.2 cells. Neutralizing antibody against mouse OSM (AF-495-SP, R&D Systems) was added to the incubation medium of C17.2 cells. After 30 min, methylmercury was further added to the medium and incubated for 24 h. Cell viability was measured by alamerBlue assay. The value of non-treated control cells was estimated as 100%. All values are represented as mean ± S.D. (n=3). $^{*}P<0.05$ vs control IgG, $^{**}P<0.01$ vs control IgG.
